# Corticothalamic Projections Gate Alpha Rhythms in the Pulvinar

**DOI:** 10.3389/fncel.2021.787170

**Published:** 2021-12-06

**Authors:** Nelson Cortes, Reza Abbas Farishta, Hugo J. Ladret, Christian Casanova

**Affiliations:** ^1^Laboratoire des Neurosciences de la Vision, École d’optométrie, Université de Montréal, Montreal, QC, Canada; ^2^Institut de Neurosciences de la Timone, UMR 7289, CNRS and Aix-Marseille Université, Marseille, France

**Keywords:** pulvinar, corticothalamic projections, alpha rhythm, cortical oscillations, cortical and subcortical loops, mathematical modeling

## Abstract

Two types of corticothalamic (CT) terminals reach the pulvinar nucleus of the thalamus, and their distribution varies according to the hierarchical level of the cortical area they originate from. While type 2 terminals are more abundant at lower hierarchical levels, terminals from higher cortical areas mostly exhibit type 1 axons. Such terminals also evoke different excitatory postsynaptic potential dynamic profiles, presenting facilitation for type 1 and depression for type 2. As the pulvinar is involved in the oscillatory regulation between intercortical areas, fundamental questions about the role of these different terminal types in the neuronal communication throughout the cortical hierarchy are yielded. Our theoretical results support that the co-action of the two types of terminals produces different oscillatory rhythms in pulvinar neurons. More precisely, terminal types 1 and 2 produce alpha-band oscillations at a specific range of connectivity weights. Such oscillatory activity is generated by an unstable transition of the balanced state network’s properties that it is found between the quiescent state and the stable asynchronous spike response state. While CT projections from areas 17 and 21a are arranged in the model as the empirical proportion of terminal types 1 and 2, the actions of these two cortical connections are antagonistic. As area 17 generates low-band oscillatory activity, cortical area 21a shifts pulvinar responses to stable asynchronous spiking activity and vice versa when area 17 produces an asynchronous state. To further investigate such oscillatory effects through corticothalamo-cortical projections, the transthalamic pathway, we created a cortical feedforward network of two cortical areas, 17 and 21a, with CT connections to a pulvinar-like network with two cortico-recipient compartments. With this model, the transthalamic pathway propagates alpha waves from the pulvinar to area 21a. This oscillatory transfer ceases when reciprocal connections from area 21a reach the pulvinar, closing the CT loop. Taken together, results of our model suggest that the pulvinar shows a bi-stable spiking activity, oscillatory or regular asynchronous spiking, whose responses are gated by the different activation of cortico-pulvinar projections from lower to higher-order areas such as areas 17 and 21a.

## Introduction

Integrating different visual attributes of an image into a single neuronal representation is a difficult task. Throughout evolution, the mammalian visual cortex has solved this computational problem by separating these different features into distinct and parallel processing modules (Bullier, 2001). Interactions between these modules are hierarchical; as more complex levels of organization are created from lower ones (Felleman and Van Essen, 1991; Bullier, 2003; Hegde and Felleman, 2007). This feedforward pathway is accompanied by feedback projections that shape responses to those upcoming signals. Thus, visual processing consists of cortical signals traveling from lower to higher order (HO) areas and vice versa throughout cortico-cortical connections, whose organization follows a hierarchical pattern. This anatomical and functional arrangement of cortical visual areas connected through specific laminar projections is the core of the cortico-centric view of visual integration (Nassi and Callaway, 2009).

Besides direct communication of cortico-cortical areas *via* feedforward and feedback connections, indirect communication through cortico-thalamo-cortical connections also occurs. These transthalamic pathways enable communication between all cortical areas through a limited number of synapses (putatively, only one) in higher order (HO) thalamic nuclei (Sherman and Guillery, 2002; Casanova, 2003). In the visual system of large mammals, the pulvinar is the most prominent HO nuclei and it establishes reciprocal connections with virtually all visual cortical areas of the neocortex (Shipp, 2003; Sherman and Guillery, 2011). This connectivity is reflected in the response properties of pulvinar neurons, which resembles those found in visual cortical cells at different hierarchical levels (Bender, 1983; Casanova, 2003, 2021; Le et al., 2019). It has been recently suggested that the unique network created between the pulvinar and the cortex is used to mediate the temporality of cortical communication (Saalmann et al., 2012; Fiebelkorn et al., 2019; Cortes et al., 2020). For instance, the pulvinar may regulate cortical responses in feedforward and feedback directions by synchronizing distant oscillatory cortical regions, given its strategic position within the visual hierarchy. Such temporal control may be crucial for shaping whole-brain dynamics on a moment-to-moment basis when, for example, attention demand or visual contrast modulation is required (Shipp, 2004; Snow et al., 2009; Cortes and van Vreeswijk, 2012; Cortes et al., 2020; de Souza et al., 2020).

**FIGURE 1:**
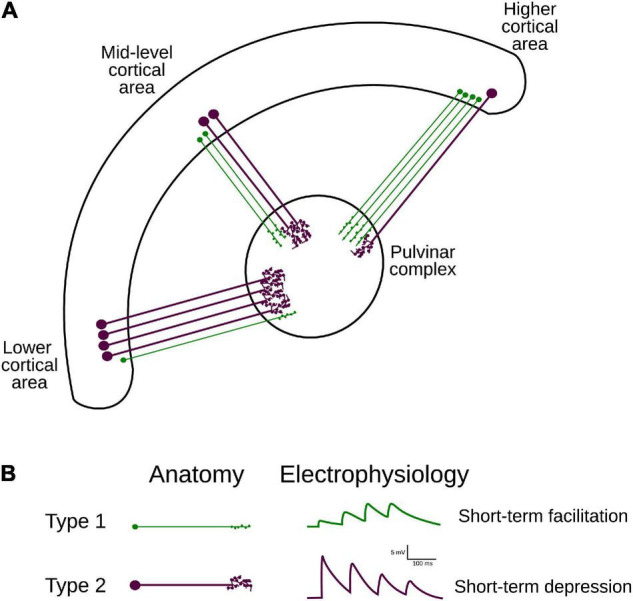
Hierarchical organization and attributes of CT projections. **(A)** Two types of CT projections from lower- mid- and higher-ranked cortical areas target the pulvinar. Note the variation of proportions of type 1 (from layer VI) and 2 (from layer V) axons along the visual cortical hierarchy (Table 1). **(B)** Anatomical (left panel) and functional (right panel) classification criteria for projection types 1 and 2. Type 1 terminals have thinner axons, bear small boutons, and elicit EPSP with short-term facilitation; type II projections have thicker axons, larger boutons that can form rosette-like structures and produce EPSP with short-term depression.

Although the transthalamic pathway is now considered as an essential part of the visual system, how visual processing along this indirect cortical route differs from that along the cortico-cortical pathways has remained elusive (Casanova, 2003). Efforts have been made to determine the anatomo-functional characteristics of the projections between the cortex and the pulvinar. Two types of corticothalamic (CT) projections have been recognized in thalamic nuclei, including the pulvinar. Type 1 axons are thin and have long, thin branches with small terminal endings and are considered to be equivalent to round small (RS) presynaptic terminals observed at ultrastructural level (Rockland, 2019); type 2 axons have thicker axon diameters with clustered endings considered to be equivalent to round large (RL) presynaptic terminals. These terminals originate in different layers: while type 1 projections arise from layer 6, type 2 projections from layer 5 (Vidnyánszky et al., 1994; Ojima et al., 1996; Feig and Harting, 1998; Huppe-Gourgues et al., 2006, 2019). In addition, based on the characterization of their excitatory postsynaptic potentials (EPSPs), type 1 and 2 CT projections display frequency-dependent facilitation and depression, respectively (Li et al., 2003). While type 1 terminals are associated with CT projections from area 17 to LGN, type 2 terminals are more abundant in CT projections from area 17 to the pulvinar (Abbas-Farishta et al., 2020). Altogether, these findings suggest that the types 1 and 2 associated pathways appear to complement synergistically each other to fine-tune visual processing passing by the pulvinar (Figure 1).

Although the pulvinar receives more type 2 than type 1 axon terminals from area 17, this ratio is not fixed within the visual cortex. Type 1 endings seem to be more represented in CT connections from higher hierarchical levels (Abbas-Farishta et al., 2020). For instance, in cats, CT terminals emerging from HO cortical areas, as areas 21a [considered to be a homolog of primate area V4 (Payne, 1993)] and the posteromedial lateral cortex (PMLS, the homolog of area MT in primates (Payne, 1993; Huppe-Gourgues et al., 2019), display more type 1 terminals. Furthermore, in the anterior ectosylvian visual area (AEV), one of the highest areas in the hierarchical organization of the visual system, the proportion of type 1 endings highly dominates the CT pathway toward the pulvinar (Table 1). These findings indicate that the ratio of type 1/type 2 cortico-pulvinar projections increases as a function of the hierarchical position of the source cortical area.

**TABLE 1:** Percentage of CT terminal types as a function of cortical source for areas 17, PMLS, 21a and AEV in cats.

Area	Type 1 (%)	Type 2 (%)
17	25	75
21a	81	19
PMLS	71	29
AEV*	91	9

Terminals located in the pulvinar subdivision Lateral Posterior lateral (LPl), except for * which comes from Lateral Posterior medial (LPm) (Abbas-Farishta et al., 2020).

This organizational scheme of CT terminals raises questions about their function and, consequently, the role that pulvinar might play in transthalamic cortical communication. On the one hand, theoretical works have shown that a population of neurons receiving type 1 and 2 terminals exhibit rhythmic or regular firing rate responses given the short-term plasticity dynamics that those terminals have (Tsodyks et al., 1998). On the other, as experimental data shows, irregular spiking activity (Chalupa, 1991; Yu et al., 2018; Wilke et al., 2009) and low-oscillatory rhythms (Saalmann et al., 2012; Fiebelkorn et al., 2019; Halgren et al., 2019) have been detected in the pulvinar. Within the slow rhythms, the alpha-band oscillations (7.5–12.5 Hz) associated with thalamic activity are particularly interesting, as animals performing visual attentional tasks show that the pulvinar drives cortical alpha rhythms (Saalmann et al., 2012), and that cortical alpha waves amplitude decreases when the pulvinar is inhibited (Zhou et al., 2016). One might ask whether the hierarchical gradient of CT terminal types 1 and 2 toward the pulvinar is responsible for generating rhythmic or irregular spiking responses (asynchronous state) in the thalamus. Therefore, to investigate whether CT projection types along the visual hierarchy influence pulvinar neuronal temporal responses differently, we simulated a pulvinar-like network of excitatory and inhibitory neurons receiving terminal types 1 and 2 from two cortical structures simulating a low and a higher-ranked cortical area. The distribution of these corticopulvinar terminals was established using projection patterns of area 17 and extra-striate area 21a of cats, whose anatomy and functional connectivity with the pulvinar have been well documented (Abbas-Farishta et al., 2020, 2021; Cortes et al., 2020; de Souza et al., 2020, 2021). Corticopulvinar projections were implemented with short-term plasticity dynamics, in which terminal types 1 and 2 had facilitation and depression of their EPSP, respectively (Li et al., 2003). Thus, connections were established to reproduce alpha-band oscillations in pulvinar neurons’ populations. We found that alpha rhythms in the pulvinar are generated by each cortical area separately or by the simultaneous combination of the two areas in a specific range of connectivity weights. In the first case, when each cortical area independently evokes pulvinar alpha waves, the oscillatory low-frequency activity generated by one area was changed by asynchronous spiking activity when the other targets the pulvinar. This property suggests that the pulvinar has a bi-stable state of oscillatory or asynchronous responses depending on the origin of the activation coming from CT afferent projections along the visual cortical hierarchy.

## Materials and Methods

### Network Models

**FIGURE 2:**
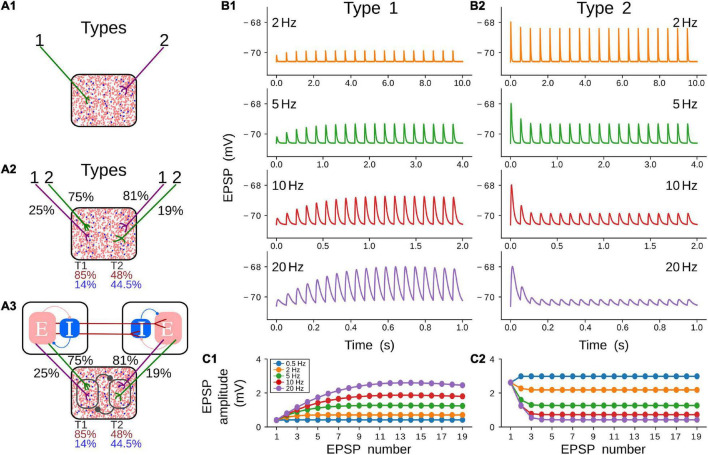
Models and short-term plasticity for synapses used in this work. **(A)** Three models were used during simulations. All models contain a pulvinar structure with 80% of excitatory (red dots) and 20 % of inhibitory (blue dots) neurons as represented in the proportion of dots inside boxes. **(A1)** Pulvinar receives only terminal types 1 and 2. **(A2)** Two areas with proportion and number of contacts of terminal types 1 and 2 in areas 17 and 21a as described in Tables 1, 2. **(A3)** Cortico-pulvinar-cortical model consisting of three networks connected feedforwardly by cortico-cortical pathways and with a transthalamic pathway. A similar proportion of connections as in panel **(A2)** was used. **(B)** Short-term plasticity for a pulvinar neuron receiving terminal types 1 **(B1)** and 2 **(B2)**. Four input frequencies are tested (2, 5, 10, 20 Hz) for each terminal type. **(C)** EPSP amplitude (mV) as a function of repetitions for five frequency-dependent stimulation regimes and the two types of cortical terminals shown in **(C1,C2)**. Note that the result of the stimulation at 0.5 Hz (blue line) is shown here but not plotted in panel **(B)**.

Three models were used in this work to simulate the neuronal pulvinar dynamics evoked by cortico-pulvinar projections (Figure 2A). Each model is an upgrade of the previous one to provide an integrative framework in which the role of CT projections was investigated. The first model consists of a network in the balanced excitatory-inhibitory (*E-I*) state, in which inputs are modeled as Poisson spike trains. Such inputs have types 1 and 2 dynamics, inducing Short-Term Facilitation (STF) and Short-Term Depression (STD) responses, respectively (Figures 2B,C) (Li et al., 2013). With this model, the proportion of input synapses and their strength were varied to investigate how this network, our “protopulvinar,” evoked either oscillatory or asynchronous responses. The second model is similar to the previous. However, here, the “pulvinar” network was targeted by two simulated cortical areas, each of them with a combination of type 1 and 2 synapses. As in the previous model, cells were simulated as Poisson spike trains (Figure 2A2). The proportion of type 1 and 2 projections, as well as the synaptic contact of those projections to *E-I* pulvinar neurons, were determined by available empirical data from the anatomy of cortical-pulvinar cat projections (Tables 1, 2). This model was useful to identify activation dynamic ranges of thalamic neuron populations as cortical projections reach them.

**TABLE 2:** Percentage of contacts from layers 5 and 6 of areas 17 and PMLS to excitatory and inhibitory cells in the pulvinar (Vidnyánszky et al., 1994).

Layer	Inhibitory (%)	Excitatory (%)	Area
5	44.5	48.0	17
5	45	55	PMLS
6	14	85	PMLS

Finally, the pulvinar network was targeted by other two independent networks that imitate cortical areas 17 and 21a (Figure 2A3). Each cortical area was organized to reach the *E-I* balanced state. A feedforward connection, from areas 17 to 21a, was established. Also, feedforward CT projections from the two cortical areas to the pulvinar-like structure were implemented. These cortical axon terminals were simulated with short-term plasticity dynamics, but synapses from the LGN to the area 17 (modeled as Poisson spike trains) and between areas 17 and 21a had linear integration of their synaptic inputs. Cortico-pulvinar projections with types 1 and 2 terminals were chosen randomly from neurons in areas 17 and 21a, and their proportion and contact to *E-I* pulvinar neurons were organized as in the previous model. This model does not consider direct feedforward connections from LGN to area 21a (Wimborne and Henry, 1992). Each area, including the pulvinar network, was simulated with *N* = 10,000 neurons (Vogels and Abbott, 2009; Cortes and van Vreeswijk, 2015; de Souza et al., 2020). From this number of neurons, 20% were inhibitory cells (Rodney et al., 2004). The effect of separated cortico-recipient zones in the pulvinar and their consequences in the response of 21a neurons were studied with this model.

### Neuron and Synaptic Dynamics

Thalamic and cortical neurons were modeled with adaptive exponential integrate-and-fire dynamics (Brette and Gerstner, 2005). This model consists of two coupled differential equations describing the leak current (linear component) and the spike generation component (exponential function). Also, an adaptive current, *w*, was added to simulate action potential adaptation. The membrane potential of a neuron (*i, A*, β) is given by:


(1) $$\begin{array}{c} C_{m}\frac{dV_{i}^{A,\beta }}{dt}=-g_{L}^{A,\beta }\left(V_{i}^{A,\beta }-V_{L}\right)+g_{L}^{A,\beta }{△}_{T}\text{exp}\left(\frac{V_{i}^{A,\beta }-V_{T}}{{△}_{T}}\right)\\ -w_{i}^{A,\beta }+I_{input,i}^{A,\beta },\\ \;\frac{dw_{i}^{A,\beta }}{dt}=\frac{a\left(V_{i}^{A,\beta }-V_{L}\right)+w_{i}^{A,\beta }}{\tau_{adapt}}, \end{array}$$

where *C*_*m*_ is the capacitance of the neuron, *V*_*L*_ is the leak reversal potential, *V*_*T*_ is the threshold and Δ*_*T*_* is the slope factor, τ_*adapt*_ is the time constant and *a* describes the level of subthreshold adaptation. Every time that the neuron *i* fires, *w* is increased by a current *b* (spike-triggered adaptation), and the membrane potential is reset to a fixed voltage, *V*_*r*_ of the neuron, *i*, which has, *A*, excitatory or inhibitory actions, and determined as cortical of thalamic component, *β*. Only excitatory neurons have adaptation current dynamics.

The input current that a neuron (*i, A, β*) receives is:


(2) $$I_{input,i}^{A,\beta }=I_{rec,i}^{A,\beta }+I_{ext,i}^{A,\beta }$$

where *I*^*A*,β^_*rec,i*_ characterizes the synaptic current from recurrent connections of each area *β*, of a neuron *i*, with *A, E* or *I*, attributes. When cortical cells are integrated in a network, the external current, *I*^*A*,β^_*ext,i*_ comprises one term if the unit comes from area 17 and two terms, if it comes from area 21a or pulvinar.

Synapses for cortical and pulvinar recurrent connections and from area 17 to 21a (feedforward pathway) are simulated as an instantaneous rise of synaptic current followed by an exponential decay.

Short-term synaptic plasticity (STP) is implemented with a phenomenological model (Stimberg et al., 2019b). This model considers synaptic release as the product of two variables, *x*_*s*_ and *u*_*s*_, where *x*_*s*_ represents the fraction of the total neurotransmitter that remains available for release, and *u*_*s*_ reflects the fraction of available resources ready for use, that is, the resources of the neurotransmitter “docked” for release by exocytosis by calcium sensors. After an action potential and the beginning of another, *u*_*s*_ decays to 0 at rate *ω_*f*_*, and *x*_*s*_ recovers to 1 at rate *ω_*d*_*, as:


(3) $$\begin{array}{c} \frac{du_{s}}{dt}=-\omega_{f}u_{s},\\ \frac{dx_{s}}{dt}=\omega_{d}\left(1-x_{s}\right). \end{array}$$

The influx of calcium in the terminal triggered by the arriving of action potentials modifies a fraction *U*_0_ of neurotransmitter resources not expected for release (1 *– u_*s*_*) to the “docked” state ready to be released (*u*_*S*_). Eventually, a release *r*_*s*_ from the fraction of *u*_*s*_ of the available neurotransmitter resources are generated, while *x*_*s*_ decreased by the same quantity, so:


(4) $$\begin{array}{c} u_{s}\leftarrow u_{s}+U_{0}\left(1-u_{s}\right),\\ r_{s}\leftarrow u_{s}x_{s},\\ x_{s}\leftarrow x_{s}-r_{s}. \end{array}$$

When a presynaptic action potential arrives, A synapses increase the *A* conductance, g^A^_STP_ in the postsynaptic neuron as g^A^_STP_ ← g^A^_STP_ + G_A0_r_s,_ where A = E, I and G_A0_ is the synaptic conductance. Only excitatory connections as short-term plasticity dynamics, given that only excitatory long-range cortico-cortical and cortico-pulvinar terminals have been described.

### Feedforward and Recurrent Connections

Recurrent and external connectivity for each structure were random with connection probability, *p*, specific to *E* and *I* populations (*p_*AI*_ = 0.1, p_*AE*_ = 0.5*). Input current is defined as $I_{\beta \leftarrow \gamma }^{A}\left(t\right)={\bar{g}}_{\beta \leftarrow \gamma }\left(t\right)\left(V_{i}^{A}-V_{\beta \leftarrow \gamma A}\right)$, where the term on the right side of the equation is the sum of all conductance from all presynaptic inputs on the neuron (*i, A, γ*), where γ is the source and β the targeting structure. In general, it is described as:


(5) $$g_{\beta \leftarrow \gamma ,i}^{A}\left(t\right)\frac{{\bar{g}}_{\beta \leftarrow \gamma }^{A}}{\tau_{syn}^{A}}\sum_{j1}^{N_{\gamma }}C_{ij}^{\beta_{A\leftarrow \gamma_{A,l}}}\sum_{k}e^{-\left(t-t_{j,k}^{\gamma }\right)/\tau_{syn}^{A}},$$

where $t_{j,k}^{\gamma }$ is the time of the *k*th action potential of the neuron (*j, γ*). For feedforward external inputs, γ can be the LGN, area 17 or the pulvinar, and β can be the pulvinar, area 17 or area 21a, and *A = E*. For the recurrent connectivity in the pulvinar and the cortex, β = γ, and *i ≠ j*. The connection matrices $C_{ij}^{\beta_{A\leftarrow \gamma_{A,l}}}$, for *A* = *E, I*, are random with probability *c*_β←γ_*K/N*_β←γ_ and $C_{ij}^{\beta_{A\leftarrow \gamma_{E,l}}}$ = otherwise. On average, neurons type *β* receive *K*_β←γ_ = *c*_β←γ_*K* presynaptic connections from γ neurons. Here, the conductance ${\bar{g}}_{\beta }$_←_*_γ_* describes the strength of the presynaptic input, which is scaled by *K* as ${\bar{g}}_{\beta \leftarrow \gamma }^{A}$ = $G_{\beta \leftarrow \gamma }^{A}/\sqrt{K}$, where $G_{\beta \leftarrow \gamma }^{A}$ is independent of K.

For background synaptic activity, a population of simulated neurons (*N*_*bckgrnd*_ = 8,000, *p*_*ANoise*_ = 0.5) where *A = E,I* as a Poisson-type spike train is applied to the pulvinar following the same synaptic dynamics of equation 5. The spike train is excitatory and activates excitatory and inhibitory neurons with a discharge rate of 0.1 sp/s.

When invoking STP for the CT pathway (*γ = areas 17 or 21a*, β = pulvinar), since conductance changes over time, the voltage integration assumes an effective synaptic weight, *g^A,STP^* β←γ rather than the static synaptic weights ${\bar{g}}_{\beta \leftarrow \gamma }^{A}.$

### Parameters

The parameters for the cell dynamics were Cm = 1 μ F/cm^2^, with conductance of leak currents of gL, E = 0.1 mS/cm^2^ and gL, I = 0.05 mS/cm^2^ for excitatory and inhibitory neurons, respectively. The other parameters that characterized the dynamic of neurons with a regular spiking are: VL = −70.6 mV, V_*T*_ = −50.4 mV and ΔT = 2 mV. The parameters for the adaptation current were a = 24 nS, b = 0.01 nA, and τ_*adapt*_ = 60 ms. For bursting V_*L*_ = V_*T*_ + 5 mV, and τ_*adapt*_ = 20 ms, a = 4 nS, and b = 0.5 nA. For each area, the synapses’ parameters were G_*E*0_ = 1.425 ms nS/cm^2^. G_*I*0_ = 1.89 ms nS/cm^2^, G_*EI*_ = 9.0 ms nS/cm^2^, G_*II*_ = 13.5 ms nS/cm^2^, G_*EE*_ = 22.5 ms pS/cm^2^, G_*IE*_ = 67.5 ms pS/cm^2^, with τ_*syn*_ = 3 ms and V_*E*_ = 0 mV and V_*I*_ = −80 mV (de Souza et al., 2020). For STP, parameters were settled to obtain similar synaptic performance from experimental data of terminal types 1 and 2 (Li et al., 2013). Therefore, synaptic release probability at rest *U*_0_^*type*1^ = 0.006 and *U*_0_^*type*2^ = 0.8; synaptic depression rates ω^*type*1^_*f*_ = 0.48 s^–1^ and ω^*type*2^_*f*_ = 2.0 s^–1^; synaptic facilitation rate ω^*type*1^_*d*_ = 1.5 s^–1^ and ω^*type*2^_*d*_ = 3.33 s^–1^; and, the synaptic conductance G_*A*0_, for A = E, I. Recurrent connectivity for each area (pulvinar, areas 17 and 21a when are modeled) is K, and the probability of connection was p_*A*_ = K_*A*_/N_*A*_, for A = E, I.

#### Variation of Pathway Connections

We used the factors *W_*FF*_* = 5 and *W_*CP*_* = 1.5 to change the weights of feedforward and cortico-pulvinar projections. These factors multiply the ratio *G*_*E*0_/*G*_*I*0_ for those entry inputs.

Simulations of network architecture and neuron equations were performed with Python version 3.2 using Brian2 simulator (Stimberg et al., 2019a). Euler’s integration was implemented using a time step of 0.05 ms. The accuracy of the results was verified by repeating simulations with smaller time steps (0.025 ms).

## Results

As stated above, three models were used to investigate the oscillatory gating generated by CT terminals in the pulvinar. The first model analyzed the effect of the combination between types 1 and 2 terminals that target excitatory and inhibitory (*E-I*) neurons, whose responses are in the balanced state (Figure 2A1). This model provided a basic approximation of the weight ranges of cortico-pulvinar connections that produced oscillatory and asynchronous neuronal responses in this network, our simulated pulvinar. The second model also consisted of external projections to a pulvinar-like structure. However, this model considered two external areas, cortical areas 17 and 21a, whose cortico-pulvinar projections contain a combination of types 1 and 2 synapses (Figure 2A2). The fraction of types 1 and 2 terminals and the percentage of synaptic contact reaching *E-I* pulvinar neurons were arranged with available empirical data (Tables 1, 2). This model allowed investigating the dynamics of pulvinar responses when the activation between cortical projections from areas 17 and 21a was temporarily deferred. While the first two networks used cortical inputs as Poisson spike trains, the third model simulated explicitly cortical neurons. Here, two similar networks of *E-I* neurons were implemented and connected feedforwardly to reproduce the interaction between areas 17 and 21a (Figure 2A3). Each cortical area targets pulvinar neurons, with the proportion and axon terminal contacts settled in the second model. Furthermore, for this model, cortico-pulvinar projections were divided in striate- and extrastriate-recipient zones to investigate the effect of different signaling pattering of the transthalamic pathway across cortical areas (Figure 2A3).

### Pulvinar EPSPs Evoked by Single CT Activation

Before analyzing the complete pulvinar network, synaptic plasticity of excitatory postsynaptic potentials (EPSPs) was simulated to obtain similar qualitatively experimental magnitudes of those found in neurons of the lateral posterior nucleus (Li et al., 2003), the homologous nucleus to the pulvinar in rats. To that end, a single CT fiber contacting a single neuron with exponential integrate-and-fire dynamics was simulated (Mat and Met, Equation 1). CT fibers were implemented with synaptic plasticity that simulates short-term facilitation (STF, Figure 2B1) and short-term depression (STD, Figure 2B2). Then, EPSPs from the pulvinar neurons were recorded as the cortical afferent fiber was stimulated with four sets of frequency impulses (0.5, 2, 5, 10, 20 Hz) (Figures 2B,C). With this set-up, the strength of the CT fiber was investigated, and experimental amplitude of pulvinar EPSPs were recovered.

Two types of CT fiber responses were generated (Figure 2B). These simulations revealed that changes of EPSP amplitudes elicited by the stimulus train at various frequencies follow experimental results of thalamic responses at a given set of synaptic parameters. For simulated type 1 CT fibers, the amplitude of EPSPs enhanced as the stimulation increased in frequency, depicting a saturation in the response after seven consecutive impulses (Figure 2C1). A contrary response was seen in type 2 CT fibers. Here, EPSP amplitudes decreases at higher frequencies, showing a constant response of amplitude after five consecutive impulses (Figure 2C2). The amplitude of pulvinar EPSPs showed a similar profile for frequency stimulation higher than 5 Hz. The phenomenon described here is known as short-term plasticity band-pass filtering, in which depression acts as a low-pass filter and facilitation as a high-pass filter (Izhikevich et al., 2003). The combination of the two synaptic components, STD and STF, generates a frequency-specific resonant output. Thus, the combination of type 1 and 2 terminals will produce a resonance with a different band frequency than that observed when each axon terminal activates pulvinar neurons independently. Taken together these results, our simulations evoked type 1 EPSPs with frequency-dependent facilitation (Figures 2B1,C1), and frequency-dependent depression for type 2 EPSPs in pulvinar neurons (Figures 2B2,C2).

### Pulvinar Network Responses Evoked by Type 1 and 2 Terminals

The next step was to study the effect of CT terminals in a population of *E-I* in the balanced state (Figure 2A1). The pulvinar was modeled with sparsely connected neurons but strong connections between *E-I* populations (van Vreeswijk and Sompolinsky, 1996). In this network, excitatory frequency-dependent types 1 and 2 terminals identified in the previous section were feedforwardly connected to excitatory and inhibitory pulvinar neurons. The weights of these external “cortical” synapses, *G*_*E*0_ and *G*_*I*0_, were invariant in time. The different responses of the pulvinar network were then analyzed when a factor η amplified the excitatory cortical pathway to *E-I* neurons. The logic of the η factor was to conserve the feedforward ratio (*G*_*E*0_/*G*_*I*0_) and only modulate the amplification of the external pathway to pulvinar neurons (Cortes and van Vreeswijk, 2015; de Souza et al., 2020). η_1_ and η_2_ were defined to amplify types 1 and 2 synaptic terminals, respectively. So, conductances for type 1 terminals, g^A^_STF_ ← g^A^_STF_ + *η*_1*_ G_A0_r_s_, and for type 2, g^A^_STD_ ← g^A^_STD_ + *η*_2*_ G_A0_r_s_, where *A* are for *E* or *I* processes.

#### Asynchronous and Synchronous Responses

**FIGURE 3:**
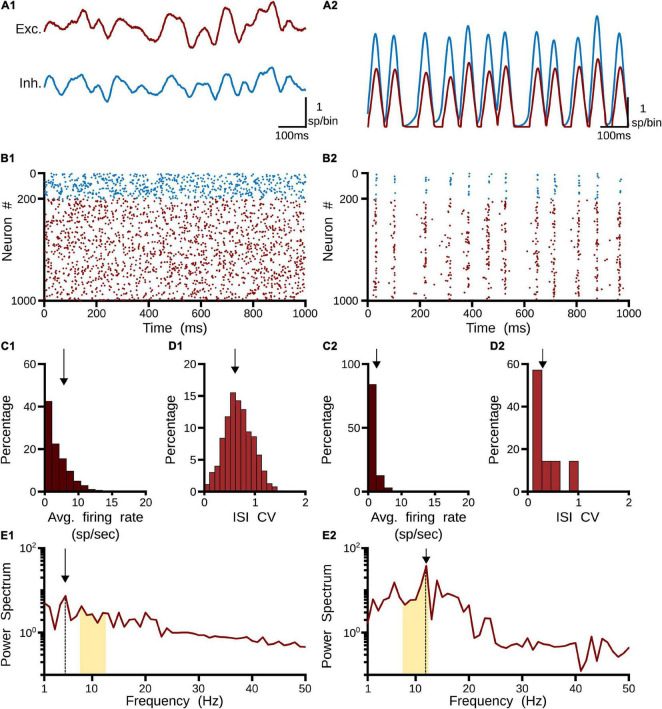
Two responses of the pulvinar network: (1) asynchronous and (2) synchronous states. **(A)** PSTH (sp/bin) of excitatory (red line) and inhibitory (blue line) neurons when a spike train with Poisson’s statistics of 10 sp/s is applied to the network, **(B)** Raster plot for excitatory (red) and inhibitory (blue) neurons. Only 1,000 cells are shown. **(C)** Distribution of average firing rate (sp/s). Arrows indicate the mean of the distribution. **(D)** Interspike intervals (ISI) of the coefficient of variation (CV). Arrows indicate the mean of the distribution. **(E)** Power spectrum of PSTHs. Arrows show the maximum frequency amplitude. Yellow zones characterize alpha-band oscillation range.

Two clear states the pulvinar network showed when η was changed: a strong irregular (asynchronous state) or a regular (synchronous state) pattern of spiking activity (Figure 3). These two activation states were evoked with a Poisson input spike train of 10 sp/s, in a network that had equal synaptic strengths to *E-I* populations of neurons. To characterize further both regimes, peristimulus-time histograms (PSTHs) and the statistics of spike discharges were analyzed. As Figure 3A shows, PSTHs for excitatory and inhibitory neurons reflect the global asynchronous (Figures 3A1,B1) and synchronous (Figures 3A2,B2) states of the pulvinar network. For the asynchronous state, spike statistics showed an exponential average firing rate and a normal distribution of the coefficient of variation (CV) for the inter-spike interval (ISI), signatures of an irregular activation regime (Figures 3C1,D1). Such asynchronous activity in recurrent *E-I* populations of neurons was obtained when the number of excitatory inputs needed to induce firing was only proportional to $\sqrt{K}$ (van Vreeswijk and Sompolinsky, 1996; Renart et al., 2010; Cortes and van Vreeswijk, 2015). This was not the case for the synchronous state (Figures 3C2,D2), in which a predominant oscillatory regime in the PSTH was revealed (Figure 3B2). Here, both the average firing rate and the ISI CV had a narrow distribution profile. These differences between the two pulvinar response states were also observed when the PSTH spectrum of frequencies was measured. In the asynchronous state, a peak of oscillatory activity was observed at low frequencies (<5 Hz), whereas, in the synchronous regime, the oscillatory activity showed a clear peak at 12 Hz, which was within the range of alpha-band oscillatory frequency. The amplitude for this peak was higher for the synchronous than the asynchronous state. In summary, the two states can be visually identified by statistical components of the firing rate and the corresponding PSTH. Thus, an asynchronous state was characterized by a high CV (close to 1) with a low amplitude frequency peak of the PSTH, while the synchronous regime had low average firing rate and CV values, and a high oscillatory frequency amplitude of its PSTH.

**FIGURE 4:**
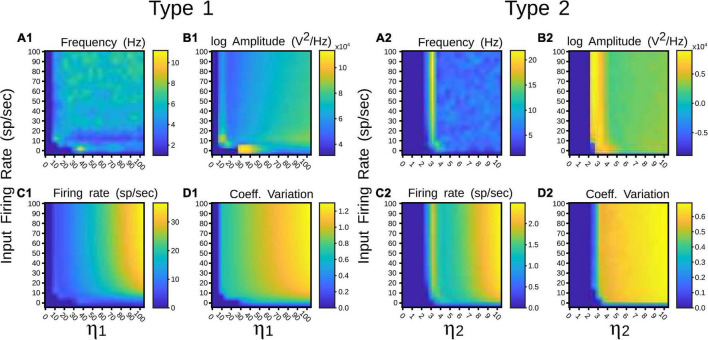
Quantitative outputs for a pulvinar network whose neurons receive terminal types 1 (1) and 2 (2) as input, and connection weights increase gradually. Factors η_1_ and η_2_ amplify corticothalamic projections of terminal types 1 and 2. **(A)** Frequency (Hz), **(B)** Amplitude (V^2^/Hz), **(C)** Firing rate (sp/s), **(D)** CV.

To identify in which set of values such neural states occurred, inputs and synaptic feedforward strengths to the pulvinar network were gradually increased. The variations were tested in two networks that had similar strengths of *E-I* connectivity. Figure 4 shows the result of such simulations. For type 1 terminals, the gradual increase of the input produced a gradual increase of the discharge of the neurons, with a clear asynchronous state of the network (Figures 4A1,B1). This gradual increase also occurred when η_1_ increased, in which the average firing rate and CV increased further (Figures 4C1,D1). Another scenario was observed for type 2 connections. Here, the pulvinar network showed a synchronous transition between the inactive and asynchronous states (η_1_ ∼3). During this transition, the firing rate as well as the oscillation amplitude increased to high value inputs, while their CV was low. Note that this oscillatory transition also occurred for type 1 synapses, but this was less pronounced. Since type 2 terminals have a first EPSP with a larger synaptic response (Figures 2B,C), these terminals evoked the oscillatory transition at lower connection intensities than those simulated for type 1 connections.

### Oscillatory Responses Evoked in the Pulvinar by Lower and Higher Cortical Areas

While the distribution of cortico-pulvinar terminal types 1 and 2 seems to be hierarchical-level-dependent, the proportion of contacts to excitatory and inhibitory neurons seem to be terminal-type-dependent. For area 17, the distribution of terminal types 1 and 2 are 25–75% respectively. In higher cortical levels, such as area 21a, this distribution is almost reversed in which types 1 and 2 are 81 and 19% of the cortico-pulvinar connections, respectively (Table 1; Abbas-Farishta et al., 2020). On the other hand, cortico-pulvinar type 1 terminals contact 85% and 14% of excitatory and inhibitory cells, respectively, type 2 synapses 48 and 44.5%, respectively (Table 2; Vidnyánszky et al., 1994). In fact, round large (RL) terminals from area 17 to the pulvinar are predominantly located in the striate recipient zone. For extrastriate cortical areas, terminal zones are characterized as small boutons (RS) (Huppe-Gourgues et al., 2006). Thus, CT projections seem to exert different synaptic actions depending on their origin along the cortical visual hierarchy and the type of terminals contacting pulvinar neurons.

**FIGURE 5:**
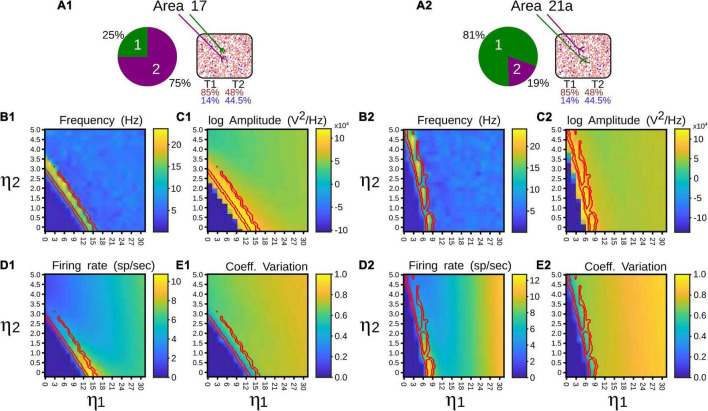
Quantitative outputs for a pulvinar network whose neurons receive projections from areas 17 (1) and 21a (2). Factors η_1_ and η_2_ amplify corticothalamic projections of terminal types 1 and 2, respectively. **(A)** Distribution and number of contacts to excitatory and inhibitory pulvinar neurons of terminal types 1 and 2 for cortical areas. Red and blue letter colors indicate excitatory and inhibitory percentages, respectively. Terminal types 1 and 2 are represented by colors green and purple, respectively. **(B)** Frequency (Hz), **(C)** Amplitude (V^2^/Hz), **(D)** Firing rate (sp/s), **(E)** CV. Red lines in panels **(B–E)** indicate zones where alpha rhythms are evoked.

Such anatomical attributes were implemented in the following simulations of the pulvinar network. For area 17, contributions from a Poisson spike train were divided in terminal types 1 and 2, with the number of implicit excitatory cells considered in the feedforward CT pathway being 25% and 75% of *K*, respectively, where *K* is the average number of total projections. For area 21a, the percentage for types 1 and 2 terminals were 81% and 29% of *K*, respectively. Regardless of their cortical area of origin, type 1 terminals had a connection probability with excitatory and inhibitory pulvinar neurons of *p* = 0.85 and *p* = 0.14, respectively, and type 2 terminals probability contacts were *p* = 0.48 and *p* = 0.445, respectively. To that network, η_1_ and η_2_ were increased gradually and input of 10 sp/s was applied to analyze the performance of the pulvinar network. The representative frequency, amplitude of this frequency, firing rate and CV were collected after 1 s of simulation, as shown in Figure 5.

#### Effect of Single Cortical Activation

Regardless of whether the input came from area 17 or 21a, at a given strength of the feedforward cortical pathway, the pulvinar network evoked oscillations in the frequency range of 7.5–12.5 Hz (alpha waves). These oscillations were generated at the transition between the quiescent and the asynchronous steady state of the network. In the transition, the network showed a maximum frequency of ∼25 Hz (beta- waves). Alpha frequency bands were found besides such maximum frequency oscillation (Figure 5, red lines). Alpha waves were symmetrical at the border of the beta bands, but for *η*_2_ high and *η*_1_ low, only one side of the transition showed alpha band responses. For areas 17 and 21a, alpha waves were around *η*_1_ = 13 and *η*_2_ = 3, and *η*_1_ = 7 and *η*_2_ = 5, respectively. Between these strengths, decreasing and increasing magnitudes of *η*_1_ and *η*_2_, respectively, allowed a continuous transition where alpha waves were always presented. Note that in order to reach such transition threshold, *η*_1_ was higher in area 17 than in area 21a. The inverse happened for *η*_2_, whose magnitude was lower in area 17 than in area 21a. Such transition threshold indicates that the proportion and the distribution of synaptic contacts of terminal types 1 and 2 settled for areas 17 and 21a promote lower values of the cortico-pulvinar connection to reach oscillatory alpha-band activity.

#### Effect on Alpha Waves of Sequential Activation of Two Cortical Areas

**FIGURE 6:**
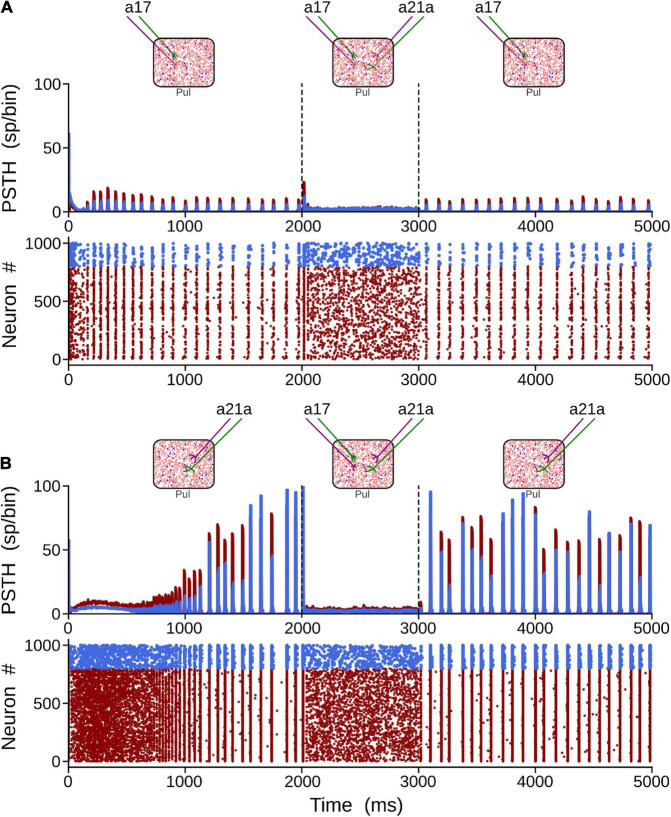
Two solutions to generate alpha waves in the pulvinar. These solutions are found when η_1_ = 0.5 and η_2_ = 2.5 for area 17, and η_1_ = 6.0 and η_2_ = 1.0 for area 21a. Note that the thalamic alpha rhythms are ceased when the other area functionally targets the pulvinar (2–3 s). Solution for areas **(A)** 17 and **(B)** 21a. Terminal types 1 and 2 are represented by colors green and purple, respectively.

Here, alpha-band oscillations generated by the driven area were measured when the other area was activated afterward. To elicit alpha rhythms that were representative of cortical areas 17 and 21a, we selected strengths of their connections such that η_2_ ≥ η_1_, and η_1_ > η_2_, respectively. In this setting, the thalamic network was simulated for 5 s. After the network reached a stable alpha-band oscillation induced by the driven area (2 s), the other cortical was “attached” (1 s). Subsequently, the attached area was disconnected, and a recovery period was allowed (2 s). The results of such simulations are shown in Figure 6. When driven inputs were from area 17, alpha waves rose quickly (∼150 ms) inside the network, having a stable oscillatory profile before the end of the first second. The amplitudes of such oscillations were low (∼15 sp/bin), even if pulvinar neurons were synchronized. In the attached period, inputs from area 21a abolished the synchronization, including alpha rhythms. Once 21a was disconnected, the pulvinar network came back rapidly to alpha-band oscillations again. When the driven input was from area 21a, the rise of alpha waves was much slower (∼1,000 ms), but amplitudes of the oscillation were much larger (∼100 sp/bin). Since type 1 synapses have low-amplitude EPSPs in their first pulses, the network takes longer to balance their inputs than when the inputs have EPSPs of high amplitude (Cortes and van Vreeswijk, 2015), as is the case with type 2 connections. Such neuronal states may be similar to those conditions found in the loss of consciousness due to analgesics (Bastos et al., 2021). Adding projections from area 17 induced an asynchronous stable activity on the thalamic population, which returned quickly to alpha waves when this attached area was disconnected. In summary, the pulvinar network developed alpha rhythms by driving cortical inputs and, at these parameter values, the arrival of other cortical sources generated a global asynchronous state and a loss of oscillatory alpha-band activity.

**FIGURE 7:**
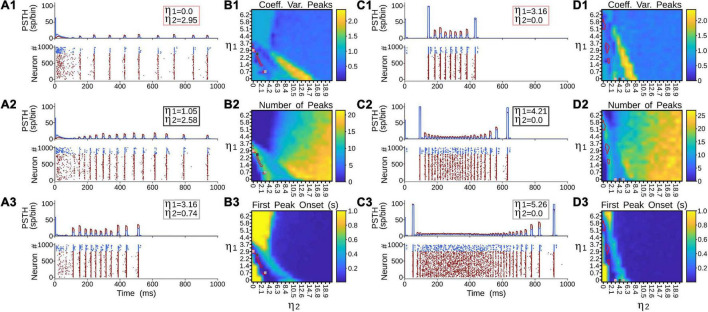
Terminal types 1 and 2 produce different activation dynamics in the thalamic network at the beginning of the stimulation. **(A)** PSTH and raster plots for area 17 solutions. **(B)** Outcomes of such solutions for (1) coefficient of variation of alpha waves peaks, (2) number of peaks per second, and (3) first peak onset. **(C)** PSTH and raster plots for area 21a solutions. **(D)** Outcomes of such solutions for (1) coefficient of variation of alpha waves peaks, (2) number of peaks per second, and (3) first peak onset.

Figure 6 showed that terminal types 1 and 2 seem to produce different activation dynamics in the thalamic network, particularly at the beginning of the stimulation. As previous pulvinar responses were only analyzed after 1 s of “recording,” the aim of the following section was to quantify pulvinar dynamics just before the onset of cortical stimulation. For this purpose, *η*_1_ and *η*_2_ were gradually increased for the strength of projections from areas 17 and 21a. The result of such iterations revealed that pulvinar alpha waves were located in similar activation zones shown above (Figure 7). In this regime, pulvinar alpha rhythms appeared and stabilized rapidly when type 2 terminals dominated cortical projections of area 17 (Figures 7A1,A2). Conversely, adding type 1 terminals and decreasing the strength of type 2 axons restricted such oscillations to a short time window (Figure 7A3). In fact, increasing the pathway strength of only type 1 axons into the pulvinar network, generated an asynchronous transition of spike activity which was subsequently transformed into a synchronous oscillation when *η*_1_ was large (Figures 7C1,C3). These qualitative details were further analyzed by measuring average dispersion (CV), number and the first-time onset of the PSTH peaks of the oscillatory pulvinar synchrony (Figures 7B–D). For area 17, alpha- waves were highly regular (low CV) when *η*_2_ > *η*_1_ (Figure 7B1), with a constant number of peaks and rapid triggering of activity in similar regimes where alpha rhythms were stable over longer simulation times (Figures 7B2,B3). Alpha rhythms for area 21a were also expressed in the same regime (i.e., *η*_2_ > *η*_1_). Here, however, when *η*_2_ < *η*_1_, alpha waves were less evident and stable oscillations with regular periodicity in the first second of simulation were undetected. Thus, in the first period of cortical stimulation, type 2 terminals were more likely to establish effectively a stable and fast alpha-band periodicity than type 1 axons regardless of their source area.

#### Simultaneous Cortical Activations

**FIGURE 8:**
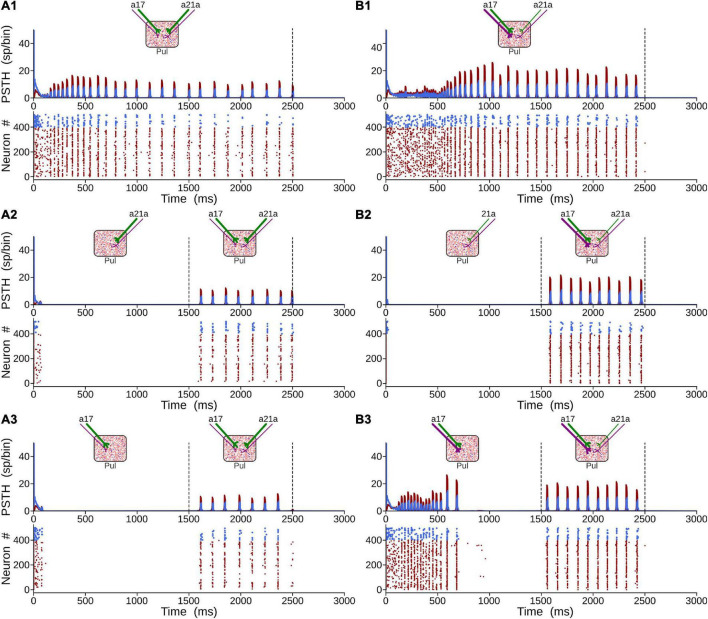
Generation of alpha waves by the combination of the two CT projections. **(A)** Solution when type 2 terminals (purple axons) are weaker than type 1 (green axons). **(B)** Solution when terminal types 1 and 2 from area 17 are stronger than connections from area 21a. For panel **(A)**, η*^a17^*_1_ = η*^a21a^*_1_ = 0.3 and η*^a17^*_2_ = η*^a21a^*_2_ = 1.8. For panel **(B)**, η*^a17^*_1_ = η*^a17^*_2_ = 2.0 and η*^a21a^*_1_ = η*^a21a^*_2_ = 0.5.

The generation of alpha waves in the pulvinar was studied when the two CT projections were combined. The objective here was to evoke alpha waves in the pulvinar with the two cortical areas activated simultaneously. This joint activation may be possible because, for instances, the superposition of oscillatory outputs from areas 17 and 21a (matrices from Figures 3B1,B2) generates spots of alpha-wave responses. To that end, the connectivity weights were iterated to find representative examples of cortico-pulvinar projections that achieved alpha rhythms in the thalamus. Such results are shown in Figure 8, in which the driver activity to pulvinar neurons was originated from the simultaneous convergence of the two cortical sources. In Figure 8A, the two cortical areas had weights of type 2 connections weaker than those from type 1, whereas, in the Figure 8B, terminal types 1 and 2 from area17 were stronger than those from area 21a. The convergence of the two cortical inputs yielded a stable oscillatory alpha-band response after 1 s, which ended when such projections were disconnected (after 2.5 s). To demonstrate that the oscillatory activity was evoked by the synergy of the two cortical areas, the thalamic network was initially connected by only one cortical area. After a period (1.5 s), the input from the other cortical area was restored (Figures 8A2,A3,B2,B3). Terminal weights used here were the same as before. In the two above cases, the input from one area was insufficient to gate oscillatory responses in the pulvinar (Figures 8A2,A3,B2,B3). When the weight of connections from area 17 to the pulvinar was more robust than those weights from area 21a (Figure 8B3), thalamic neurons showed oscillatory responses, but only during a short period (<1.0 s). In this regime of connections, alpha waves in the pulvinar recovered when connections from the disconnected cortical were added from area 17 or area 21a, showing a stable periodicity during the whole period of dual cortical stimulation.

### Oscillatory Responses of the Transthalamic Network

In this section, each cortical area was modeled explicitly as a network of *E-I* neurons whose responses were settled in the balanced state (Figure 2C3). Randomly chosen excitatory neurons formed the two CT pathways from areas 17 and 21a (*p* = 0.2, homogeneous distribution). The proportion of terminal types 1 and 2 were 25% and 75% and 81% and 29% of *Np* = *K* synapses, for areas 17 and 21a, respectively (Table 1). While the proportion of excitatory type 1 terminals contacting excitatory and inhibitory pulvinar neurons was *p* = 0.85 and *p* = 0.14, respectively, for type 2 terminals it was *p* = 0.48 and *p* = 0.445, respectively (Table 2). The two cortical areas were also connected feedforwardly through randomly chosen excitatory connections from area 17 to excitatory and inhibitory neurons of area 21a (*p* = 0.05). Inputs to area 17, from an implicit lateral geniculate thalamic nucleus (LGN), were also drawn from a uniform distribution (*p* = 0.05), and were modeled as spike trains with Poisson’s statistics. Axon terminals from LGN to area 17, and from area 17 to area 21 did not have any synaptic plasticity. The three interconnected structures mimic the cortico-thalamo-cortical network formed by areas 17 and 21a and the pulvinar.

#### Effect of Cortical and Pulvinar Dynamics in Oscillatory Responses of the Transthalamic Network

Burst discharges and low background levels of synaptic noise in the pulvinar were first settled to study the cortico-pulvinar pathway of the model. It has been postulated that intrinsic burst neurons in layer 5 (5IB) of the lower cortical areas (i.e., areas 17 and 18) participate in the conduction of pulvinar alpha rhythms (O’Reilly et al., 2021). Furthermore, thalamic bursting has been characterized as an intrinsic property of pulvinar neurons (Ramcharan et al., 2005). Besides, background noise can alter the synaptic efficiency of connections by increasing subthreshold fluctuations (Silver, 2010) and enhancing the salience of neuronal oscillations to avoid long asynchronous/synchronous state transitions as in Figures 7C1–C3. For that end, intrinsic bursting was induced in cortical and pulvinar neurons by changing mainly high reset values (*V_*r*_ > V_*T*_)*, among other parameters of the current regular spike train (Brette and Gerstner, 2005). Furthermore, stochastic balanced synaptic inputs without short-term plasticity were added equally to *E-I* pulvinar neurons to elicit background noise. Here, a simplified version of the neural network described in Figure 2C3 was used, where only CT projections from area 17 to the pulvinar were incorporated. Thus, the effect of cortical burst spikes and background noise into pulvinar neurons on thalamic alpha waves were compared to cases where no such functional biological processes were present.

**FIGURE 9:**
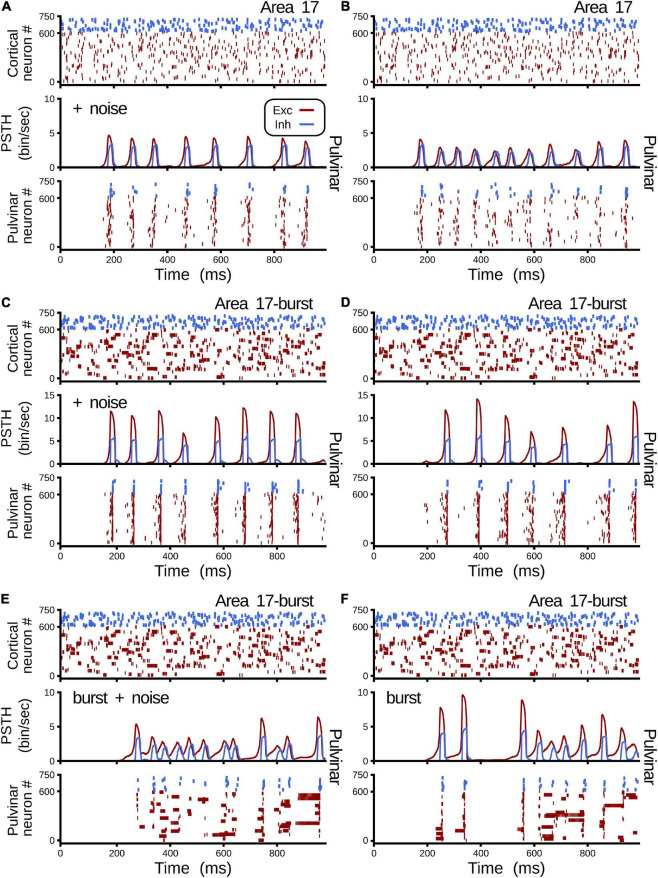
Combinations of cases when area 17 and pulvinar neurons have regular or burst discharges, and pulvinar is with or without background synaptic noise. For each case, raster plots for area 17 and pulvinar and PSTH for pulvinar are shown. Case **(A)** Regular spiking for area 17 and pulvinar neurons, background synaptic noise added to pulvinar neurons. Case **(B)** Similar to case **(A)**, but without background synaptic noise in pulvinar. Case **(C)** Bursting dynamics for area 17, regular spiking for pulvinar neurons, background synaptic noise added to pulvinar neurons. **(D)** Similar to case **(C)**, but pulvinar neurons without background synaptic noise. **(E)** Bursting dynamics for area 17 and pulvinar neurons, background synaptic noise added to pulvinar neurons. **(F)** Similar to case **(E)**, but pulvinar neurons without background synaptic noise. Supplementary Table 2 shows values of η*^a17^*_1_ and η*^a17^*_2_.

Figure 9 shows all cases of combinations where area 17 and pulvinar neurons have regular or burst spiking discharges and pulvinar is with or without background synaptic noise. Raster plots for cortical and pulvinar neurons (top and bottom panels, respectively) and PSTH envelopes for excitatory and inhibitory pulvinar neurons (middle panel) are shown here. To obtain such results, cortical area 17 was targeted by an LGN input of 50 sp/s, and cortical and pulvinar excitatory and inhibitory neurons were recorded for 1 s. Parameters of the brain structures were fixed between simulations, but cortico-pulvinar weights were fitted to obtain pulvinar alpha rhythms. Cases with background noise in the pulvinar were analyzed first, then when neurons from area 17 had bursting discharges, and finally, when thalamic neurons incorporated such burst-like dynamics. Adding background noise to pulvinar excitatory and inhibitory populations of neurons improved the profile of alpha-band oscillations (Figures 9A,B). The average dispersion of the excitatory bumps in the PSTH formed around the synchronous discharge of neurons were 30.05 ± 0.14 ms (mean ± std) and 32.54 ± 0.42 ms (*t*-test, *p-value* = 0.001) for simulations with or without background noise in the pulvinar, respectively. When cortical neurons had bursting dynamics (Figures 9C,D), bumps of alpha waves in the pulvinar with and without background, became larger with higher peaks (∼10 sp/bin) and smaller dispersion (∼29.05 ms), compared to previous cases with only regular spiking discharges (Case A vs. Case C, *t*-test *p-value = 0.06*, Case B vs. Case D, *t*-test *p-value = 0.004*). Changing pulvinar regular spiking cells with bursting neurons favored the irregular periodicity of alpha rhythms, but background noise added into excitatory and inhibitory populations stabilized partially such oscillatory lost (Figures 9E,F). Taken together, synchronous alpha rhythm responses of pulvinar neurons were affected by cortical and pulvinar neuronal dynamics as well as the background noise added to thalamic neurons.

**FIGURE 10:**
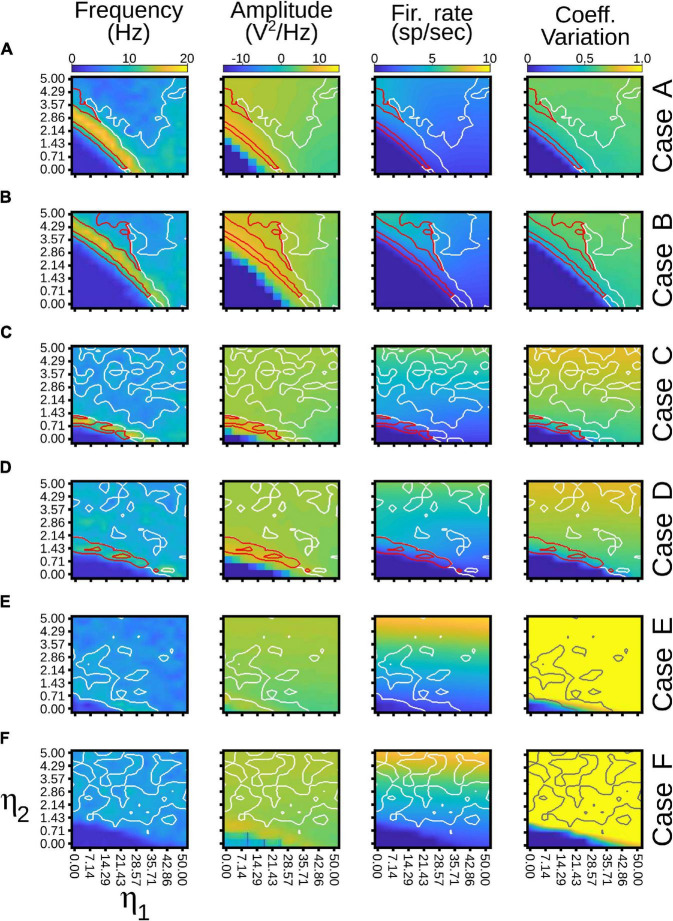
Quantitative outputs for cases in Figure 9. Frequency (Hz), amplitude (V^2^/Hz), firing rate (sp/s) and CV for different cases as η_1_ and η_2_ increase gradually. Red line, alpha waves with higher amplitude; white line, alpha waves with lower amplitude. Refer to the text for more details.

Pulvinar responses to different cortical and pulvinar dynamics were further investigated by measuring oscillatory frequency and amplitude, as well as firing rates and CV when *η*_1_ and *η*_2_ were iterated (Figure 10). Alpha waves were highlighted by depicting contours that had high and low amplitude spectral density in the Fourier transform (Figure 10, red and white lines, respectively). In general, alpha waves were between the transition of quiescent states and stable asynchronous activity. While strong alpha-band oscillations were limited to be in such transition (red lines), low energy frequencies were revealed to be more ubiquitous (white lines). Noise decreases the weights used to evoke such strong alpha-frequency oscillatory responses, when comparing iterations with and without background noise (Figure 10A vs. Figure 10B and Figure 10C vs. Figure 10D). Background noise also restricted the feasible region for finding the alpha waves. On the contrary, incorporating cortical bursting dynamics in the network expanded such regions, particularly for weak alpha-band oscillations (Figures 10B–E). Strong alpha rhythms were eliminated when burst responses were included into pulvinar neuron dynamics. As Figures 9D–E show, weak alpha waves were located at the transition from silent to activated states, when background synaptic noise was present and cortical and pulvinar neurons had bursting dynamics. Taken together, incorporating bursting cortical and pulvinar dynamics and adding background synaptic noise into pulvinar neurons enhanced oscillatory alpha-band states of the cortico-pulvinar network, but the localization of such low-frequency transition regimes was almost unchanged.

#### Effect of Transthalamic Pathway in Higher-Order Cortical Responses

Pulvinar effective connectivity to the visual cortex was tested by forming the transthalamic pathway. In this scenario, cortical area 21a received two feedforward projections: one coming from area 17 and another from the pulvinar (Figure 2C3). In turn, area 17 received an input from the LGN that consisted of spike trains with Poisson’s statistics. Connections from the LGN to area 21a were not implemented. The pulvinar was divided in two functional areas based on the arrangement of CT projections: striate- and extrastriate-recipient zones. Each recipient zone received percentage of connections and excitatory and inhibitory proportion of contacts from terminal types 1 and 2 of cortical areas 17 and 21a as previous simulations. However, connections between *E-I* pulvinar cells were strong, similar to the organization of the two cortical areas. For simplicity, only one pulvino-cortical projection was considered, which was for the connection from the pulvinar to area 21a. For this area, each excitatory and inhibitory populations received in average *K* random contacts from excitatory pulvinar neurons. Weights of this pulvino-cortical projection, *G*^21^^*a*←*pul*^_*E*0_ and *G*^21^^*a*←*pul*^_*I*0_, were multiplied by the same factor, *W*_*CP*_^21*a*^, which was fixed for all simulations. Dynamics of the pulvinar neurons used here were those established in case A (Figure 10A), when the neurons had regular spiking and low background synaptic noise. Thus, the neural network used for the next iterations consisted of a cortico-cortical feedforward pathway from area 17 to area 21a, and a transthalamic pathway from area 17 to the pulvinar, and from pulvinar to area 21a, also defined by CT projections from areas 17 and 21a to striate- and extrastriate-recipient zones in the pulvinar, respectively.

##### Propagation of Alpha Rhythms From Pulvinar to Area 21a

**FIGURE 11:**
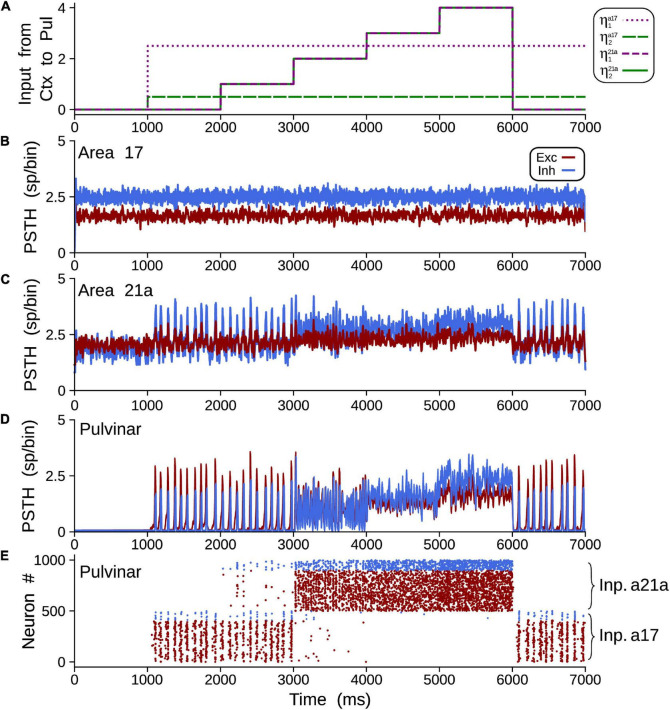
Example of oscillatory alpha-band activity transmission by transthalamic network for a network as Figure 2A3. **(A)** Weights of connections, η*^a^*^17^,^21^*^a^*_1_ and η*^a^*^17^,^21^*^a^*_2_ for areas 17 and 21a. PSTH for areas 17 **(B)**, 21a **(C)** and pulvinar **(D)**. **(E)** Raster plots for neurons in the pulvinar. Note that half of the pulvinar neurons are targeted by CT projections from area 17 and the other from area 21a.

To achieve alpha waves in the pulvinar, CT projections from area 17 to the pulvinar were fixed to a constant value, and CT weights from area 21a to the pulvinar were gradually varied. Figure 11 shows an overview of the network performance. For these simulations only, *η*^21^*^a^*_1_ = *η*^21^*^a^*_2_. During the first second, the cortico-cortical pathway was attached, whereas the cortico-thalamo-cortical pathway was unconnected. By consequence, predominant low-frequency oscillations were absent in area 21a. Then, CT from area 17 and pulvino-cortical projections to area 21a were added. In this configuration, alpha-band oscillations were generated in cortical area 21a by the transthalamic pathway (1–2 s). Note that during this period, alpha waves were only evoked in the pulvinar striate-recipient zone. In the next temporal sequence (2–6 s), CT connections from area 21a were added into the pulvinar extrastriate-recipient zone, and these projections were amplified gradually throughout this period. Alpha waves in area 21a and the pulvinar persisted when cortical projections had a low magnitude (2–3 s). In the contrary, alpha-band oscillations were lost when *η*^21^*^a^*_1_ and *η*^21^*^a^*_2_ enhanced (3–6 s). The pulvinar and area 21a showed an asynchronous profile of spike activity when cortical top-down connections to the pulvinar were sufficiently high. Note that pulvinar responses were produced only in the extrastriate-recipient zone, while responses in the striate- recipient zone were silenced. Finally, alpha waves in the pulvinar and area 21a were re-established when cortico-pulvinar connections from area 21a were disconnected (6–7 s). Taken together, the transthalamic pathway enabled functional connectivity in alpha frequency band when cortico-pulvinar connections from lower cortical areas were allowed and an asynchronous response in higher cortical areas when the cortico-pulvinar loop between area 21a and pulvinar was closed.

##### Closing the Cortico-Pulvinar Cortical Loop

**FIGURE 12:**
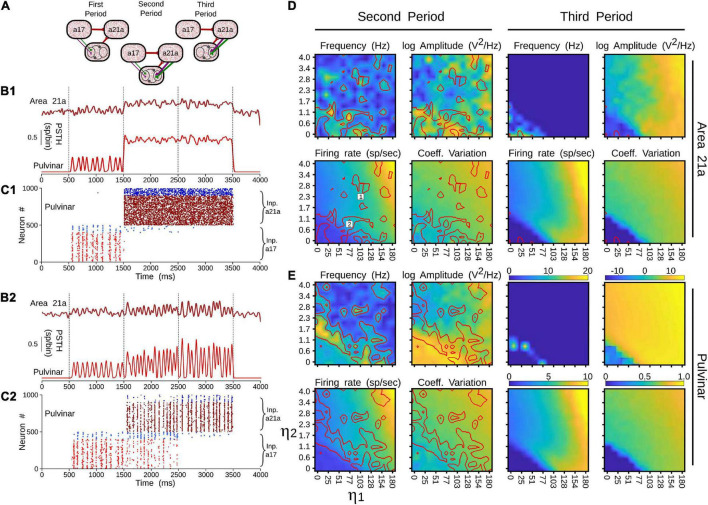
Effects on alpha waves when the loop between area 21a and the pulvinar is closed in the cortico-thalamo-cortical model. **(A)** Scheme showing the temporal configuration of the three periods for the connections of the model. Panels **(B,C)** are two solutions when top-down loop to the pulvinar generate asynchronous (1) or oscillatory (2) responses. **(B)** PSTH for area 21a and pulvinar. **(C)** Raster plots for pulvinar neurons with striate- and extrastriate-recipient zones. Second and third periods’ quantitative outputs (frequencies, amplitudes, firing rates and CVs) for area 21a **(D)** and **(E)** pulvinar networks when η^21^*^a^*_1_ and η^21^*^a^*_2_ are increased gradually. Note the position of solutions 1 and 2 in the panel **(D)**.

The effect of closing the loop between cortical area 21a and the pulvinar on the formation of alpha waves was further analyzed by varying the weights of terminal types 1 and 2 of these CT projections. For that end, a network incorporating cortico-cortical and transthalamic pathways (area 17 to pulvinar, pulvinar to area 21a) was created as above described. In addition, the network had a top-down projection from area 21a to the pulvinar, which was organized in three different time periods. In the first condition, the projection from area 21a and the pulvinar was disconnected (open loop) for 1 s (Figure 12A). The next second, the projection from area 21a to the pulvinar was formed (close loop), and connections from area 17 to the pulvinar were still activated. In the last second, only the close loop was functional, in which cortical top-down activity to the pulvinar was evoked indirectly by cortico-cortical arriving inputs to area 21a (Figure 12B).

With this network, the efficacy of alpha waves expressed in area 21a by pulvinar projections was quantified. For the first second, alpha waves in the pulvinar and area 21a were evoked 99% and 64.44% of the time, respectively. The remaining ∼35% of oscillations evoked in area 21a were lower than 7.5 Hz. In the next condition, *η*^21^*^a^*_1_ and *η*^21^*^a^*_2_ were iterated and the frequencies, amplitudes, firing rates and CV of area 21a and pulvinar outputs were characterized. Figure 12C shows that, in the second period (2–3 s), closing the loop between area 21a and the pulvinar decreased alpha-band oscillations in the two networks. In fact, the pulvinar achieved only 36% of alpha waves, causing a loss of alpha-wave transmission in area 21a (∼26%). Alpha-band oscillations in the pulvinar were only expressed at low magnitudes of *η*^21^*^a^*_1_ and *η*^21^*^a^*_2_, since the striate-recipient zone continued to yield such rhythms. When the strength of the cortico-thalamo projections, from area 21a to the pulvinar extrastriate-recipient zone was increased slightly more, the striate-recipient zone was perturbed, and so was the production of alpha waves, decreasing the propagation of such frequency into area 21a during this second period. Note that a variety of higher frequency oscillations in area 21a and the pulvinar arose at such intermediate neuronal states (Figure 12B1). As the strengths of the CT connections were higher, only extrastriate-recipient neuronal responses in the pulvinar were engaged, causing an asynchronous spiking state in both the pulvinar and area 21a (Figure 12B2). Similar responses were observed in the last period of stimulation (3–4 s). Here, the closed loop was still coupled, but connections from area 17 to the pulvinar were disconnected. Under this configuration, only 1.7% of alpha waves were generated in area 21a. In average, the pulvinar did not show any alpha waves during this period. The remaining spiking responses of area 21a and the pulvinar had almost all asynchronous features or higher-frequency oscillations. More details of the activity throughout these periods are shown in Supplementary Table 1. Altogether, similar to previous analysis, the transthalamic pathway allowed a propagation of alpha waves from the pulvinar to area 21a, which was occluded by the arriving of the top-down projection from area 21a to the pulvinar. Thus, the pulvinar showed a bi-stable spiking response, oscillatory or irregular spiking, whose responses were gated by the different activation of projections from areas 17 and 21a to the pulvinar.

## Discussion

Previous experimental studies have shown that neuronal populations of the pulvinar nucleus possess multiple dynamic activity profiles (Wrobel et al., 2007; Saalmann and Kastner, 2011; Yu et al., 2018; Le et al., 2019). In this work, using a theoretical approach, we propose that such population dynamics in the pulvinar may arise from CT connections originating from different hierarchical cortical areas, whose axonal terminals have distinct anatomical and physiological profiles. Two types of neuronal discharges were evoked in the pulvinar, synchronous and asynchronous responses, when projections from areas 17 and 21a to the pulvinar were organized according to the connection empirically described in cats (Huppe-Gourgues et al., 2006, 2019; Abbas-Farishta et al., 2020). We found that, at a specific connection weight regime, CT connections reproduce the oscillatory alpha-band activity of the pulvinar. This solution from the model was found when each cortical area independently contacted the pulvinar and when they were combined. When a single area evoked oscillatory alpha-band activity in the pulvinar, the activation of the distant area ceased such oscillations. The combinations of the two projections types 1 and 2 changed the oscillatory dynamics to asynchronous spiking states. Similar bi-stable state has been reported experimentally in the primate pulvinar during object detection and passive viewing (Wilke et al., 2009). The results found in our models are a direct consequence of the restrictions we put on the parameters, suggesting that the pulvinar may have several dynamic response states to interact with the visual cortex.

The most important prediction of this work is that the pulvinar has at least two functional response states, regular oscillatory or stable asynchronous. Other models explaining such oscillatory variations have included alpha waves in the pulvinar implicitly (Quax et al., 2017) or only as part of the cortico-cortical circuitry (Jaramillo et al., 2019). Our model suggests that part of such oscillatory activity comes from types 1 and 2 CT projections distributed as a gradient along the hierarchy of the visual system. While alpha rhythms in the pulvinar may reflect the functional cortical feedback connectivity (Halgren et al., 2019), our work is in agreement with the experimental evidence that the pulvinar drives cortical alpha waves (Saalmann et al., 2012). In addition, our model predicts that such oscillations may be abolished by CT projections from lower cortical areas (areas 17 and 18). As shown here, given that such connections have a high proportion of type 2 connections, their role would be to desynchronize the pulvinar when activated in a synchronous manner. Although, additional anatomical and physiological data are needed to confirm our hypothesis, the implications of such predictions are explained in more detail below.

### Mechanism of Generating Oscillations

To obtain oscillatory alpha-band activity, in our model, we settled connections to be unbalanced, so CT weights were just lower than $\sqrt{K}$ synapses providing low-frequency waves. Since oscillatory solutions were in the frontier of a balanced state (Figure 6), the addition of an extra input generated an asynchronous irregular spiking activity. Such a neural property of networks in the balanced state explains the sharp transition between the two states, which could have implications for fast temporal neural encodings in the pulvinar, as recently proposed (O’Reilly et al., 2021).

Mechanisms that generate alpha rhythms have been attributed to collateral branches of CT axons to the thalamic reticular nucleus (TRN), which in turn provide inhibitory inputs back to the thalamus. Such glutamatergic excitatory input from cortical layer VI targets excitatory and inhibitory TRN cells, while the latter component, GABAergic neurons, hyperpolarized thalamic excitatory relay cells that discharge action potentials in burst firing rate. The burst stimulates collateral TRN cells and serves to re-inhibit excitatory relay cells. This loop continues in a low-frequency oscillation at 7–14 Hz (Jones, 2002; Crabtree, 2018). Our model postulates a different mechanism for generating alpha rhythms from the balanced *E-I* networks’ intrinsic properties (see above) and the oscillatory property evoked by the interplay of short-term plasticity components. The combination of facilitatory and depression synapses can generate time-dependent connectivity effects, which engage pulvinar neurons to oscillate in specific low-band frequencies. Such resonant activation acts as a band-pass filter, eliciting a specific amount of neuronal information and communication between the cortex and the thalamus (Markram et al., 1998; O’Brien et al., 2014). The model, parameterized with the experimental proportion of CT terminal types 1 and 2 of two cortical areas, suggests a possible non-GABA mediated mechanism as suggested by recent pulvinar causal manipulation results in monkey (Saalmann et al., 2012; Zhou et al., 2016).

In a more natural scenario, the addition of noise and other membrane dynamics favored the appearance of more realistic scenarios similar to previous experimental works. The addition of a synaptic noise background (Figure 9A) allowed decreasing the delay to reach this oscillatory stability (Silver, 2010). The addition of burst-like dynamics favored irregular oscillations and lower power in the amplitude of the signal (Ramcharan et al., 2005; O’Reilly et al., 2021). However, when the cortico-pulvino-cortical model was implemented, we selected pulvinar neurons with regular spiking responses with a low background synaptic noise (case A, Figures 10, 11). Such properties were chosen to reinforce the regular oscillatory activity of the pulvinar on the neural responses of the visual cortex. Although the burst-like responses may refine the neuronal responses of the pulvinar to a more realistic scenario, our model predicts that such stable oscillatory responses are due to the physiological properties of terminal types 1 and 2 and how these are combined to obtain a nearly balanced state.

### Implications in the Transmission of Oscillatory Cortical Responses Throughout the Transthalamic Pathway

**FIGURE 13:**
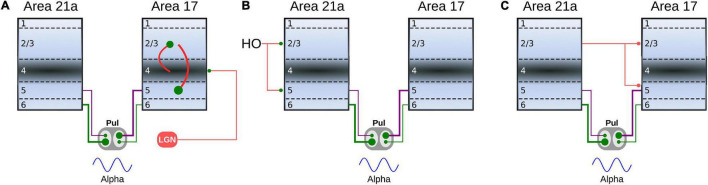
Schematic representation of the oscillatory responses predicted by our models. **(A)** The pulvinar evokes an alpha-band oscillatory response by the feedforward activity of area 17. The response of the infragranular layers of area 17 is evoked by the inter-laminar connectivity that initially reaches layer 4. **(B)** Oscillatory alpha-band responses in the pulvinar are evoked by feedback activity from higher order (HO) cortical areas to the infragranular layers of area 21a. **(C)** Indirect oscillatory alpha-band activation of the pulvinar by the feedback from area 21a to the infragranular layers of area 17.

Visual processing in cortical areas follows mostly a hierarchical stream of communication, in which the visual information travels from low to high levels (feedforward pathway), as well as from high to low levels (feedback pathway) (Felleman and Van Essen, 1991; Bullier, 2003). Oscillatory-band activity has been associated with these anatomical pathways, in which gamma-band oscillations are found in the feedforward route, whereas slow oscillations, alpha/beta waves, are observed in the feedback direction (van Kerkoerle et al., 2014; Bastos et al., 2015). In our simulations, nonetheless, we show that the alpha-type oscillatory activity evoked in area 21a, we are able to reverse that transmission in feedback action if η^21^*^a^*_1_ > *η*^21^*^a^*_2_, *so* reversing the ratio of types 1 and 2 terminals, and introducing a pulvino-cortical projection such as *W*_*CP*_^*a*17^< *0*. In this new scenario, CT projections from area 21a would drive alpha-band oscillatory responses in the pulvinar, whereas CT connections from area 17 to the pulvinar would shift such responses into asynchronous-type activity (Figure 13).

Reversing the direction of transmission along the cortical hierarchy, and changing the activity from a synchronized to an unsynchronized state, depends on two properties of the simulated network. As described above, terminal types 1 and 2 can evoke alpha-band oscillations independently. Interestingly, as the terminals are set with the ratios of areas 17 and 21a, solutions are found when the predominant terminal had a higher weight of connection than the other type. That is, to engage alpha waves in the pulvinar by CT projections from area 17, the type 2 terminal had to be stronger than the type 1, and vice versa for area 21a, which follows the ratios found in the empirical anatomical data. The second property is derived from the two compartments organized in the pulvinar as striate- and extrastriate-recipient zones. Our model predicts that activity between these zones would compete with each other for activation in the pulvinar, similar to a “Winner-Take all” (WTA) process. Since these zones are activated independently by different cortical areas along the visual hierarchy, asynchronous or synchronous activity within the pulvinar will also be triggered independently. Such hypothetical compartmentalization of responses predicted by the model allows the pulvinar to revert its oscillatory alpha-band activity to an asynchronous irregular spiking or vice versa as shown by recent experimental data (see below). Thus, the pulvinar filters one type of neural response over the other, depending on which cortical area projects more strongly to it.

The pulvinar, across different species, is a heterogeneous structure that is composed of multiple subdivisions and, unlike the LGN, such separations have little organizational arrangements like neuronal lamellae (Baldwin and Bourne, 2017). These anatomical features mean that defining the function of the pulvinar throughout mammalian evolution remains a challenge (Casanova, 2003). Our model, with at least two-separated cortical-recipient zones, may clarify functional aspects that the pulvinar has. As our model predicts, different low-band frequencies are built in the pulvinar as the strength of CT connections increases gradually, mainly when type 1 connections are used (Figures 4, 11). If several cortical areas are represented independently as separate connections domains in the pulvinar (Shipp, 2003), these pulvinar domains could generate different oscillations regulating visual cortical activity and coordinating transthalamic messages in an oscillatory ascending manner to the visual cortex. Thus, like a WTA computation, the pulvinar would serve as a channel selector of different band frequencies that would adjust cortical dynamics to be able to transmit oscillatory low-frequency activity from one group of neurons to another, possibly in a feedforward or feedback manner throughout the visual hierarchy (Quax et al., 2017; Jaramillo et al., 2019; Cortes et al., 2020). In other words, the pulvinar could select and separate signals from and to the visual cortex by the above-described WTA mechanism. According to this view, the pulvinar would use two anatomical properties to allow visual processing in the cortex: a gradient of terminal types 1 and 2 throughout the cortical hierarchy (Abbas-Farishta et al., 2020), and the spatial processing by the compartmentalization of cortical-recipient zones suggested by our model. Therefore, the pulvinar would need a differential increase in type 1 terminal weights along higher order visual cortical areas and cortico-recipient zones that are partially isolated to carry out this selection mechanism (Huppe-Gourgues et al., 2006, 2019; Abbas-Farishta et al., 2020).

The addition of more inputs into the pulvinar would explain the appearance of other low-frequency oscillations observed in *in vivo* experiments. As our simulations predict, the increase of strengths of the CT projection from area 21a (Figures 3, 11) causes a shift to other oscillatory frequencies by the competition between rival cortical-recipient zones in the pulvinar. One interpretation of these results may be the recruitment of other higher cortical areas increasing the number of CT type 1 endings along the visual cortex that reach and activate synergistically the pulvinar. According to this view, *η_1_* in our simulations is not static; it changes dynamically throughout the visual cortical hierarchy to meet behavioral demands matching cognitive states (i.e., attentional demands). As more and more areas project to the pulvinar, this top-down activation could generate different band frequencies, which is seen for theta-, beta- and gamma-band oscillations in awake animals (Wrobel et al., 2007; Saalmann and Kastner, 2011; Yu et al., 2018). On the one hand, the pulvinar may maintain specific resonant frequencies with specific cortical areas that allow cortico-cortical feedforward and feedback processing to propagate in one direction or the other (Saalmann et al., 2012; Zhou et al., 2016; Cortes et al., 2020). Another simple interpretation of alpha rhythms in the pulvinar are not optimized for coupling between cortical areas, as areas 17 and 21a, since one cortical area would dominate its representation inside the pulvinar over the others. Future experiments will reveal what mechanism the pulvinar uses to maintain its functional coherence with the visual cortex.

On the other hand, the oscillatory response provoked by the joint action of the two areas (Figure 8) could also be interpreted as a convergent action of connections coming from the visual cortex and other brain structures. As our model shows, we found a solution to evoke alpha rhythms when areas 17 and 21a had similar weights of connections. Since the connectivity weights of the cortical areas across the visual hierarchy are unlikely to be similar (because of their different physiological characteristics), one prediction of the model is that one of those connections is not from the visual cortex. Instead, it would arise from subcortical projections, for example, from the superior colliculus. This joint cortical and subcortical action could engage pulvinar responses in different oscillatory modes (Le et al., 2019), when, for example, eye movements are required (Berman and Wurtz, 2011). Thus, the recruitment of other cortical areas and the combination with other subcortical structures could explain the different oscillatory ranges found in the pulvinar.

Another prediction of our model and the WTA proposed mechanism is that the inhibition of one compartment in the pulvinar can increase the activity in another cortico-recipient zone. For example, in Figure 12, stopping the extrastriate-recipient zone’s activity produces excitation of the striate-recipient zone, which causes an increase of alpha-band oscillations in area 21a by the transthalamic pathway. This excitatory oscillatory effect on cortical populations of neurons when local inhibitions are made in restricted areas of the pulvinar has been already quantified experimentally (Cortes et al., 2020). Alternatively, this antagonistic effect could also explain the effect of GABA inactivation in the pulvinar, in which the firing rate of cortical neurons achieves a reduction and an enhancement in areas 17 and 21a of cats, respectively (de Souza et al., 2020). Likewise, pulvinar inactivation potentiates both stimulus-driven responses in monkey V2 cells (Soares et al., 2004) and low-frequency LFP power during monkey visual attentional tasks (Zhou et al., 2016). Results that point in the same direction that the suppressive effects of the surrounding regions of V1 receptive fields when pulvinar is excited (Purushothaman et al., 2012). The suggested compartmentalization of the pulvinar would explain why disruption of the activity of isolated domains could create excitation of other nearby domains within the pulvinar.

### Implications of Pulvinar Bistable States

Perception of low contrast stimuli reveals that the brain shows two temporal states of visual awareness processing (van Vugt et al., 2018). The brain processes external stimuli in a modular and parallel bottom-up hierarchical fashion if the stimulus is strong enough and exceeds an internal threshold of visual perception. This first state is essentially feedforward. Such signals are retrieved and selected in a second state through attentional requirements located in hierarchically elevated cortical areas. Here, the flow pathway is in a feedback manner, favoring convergence to activate necessarily and sufficiently all the network nodes (Dehaene et al., 2011).

Although both cortical states have been explained based on the long-range excitatory cortical connection, we postulate that the pulvinar is involved in changing from one state to another. Such control would be possible because of the bi-state generated by the hierarchical gradient of CT terminal types 1 and 2 reaching the pulvinar. Theoretically, it has been shown that the pulvinar and its transthalamic pathway are necessary to pass neural responses in a graded manner through a chain of sequentially connected areas (Cortes and van Vreeswijk, 2012, 2015; de Souza et al., 2020). Such a prediction of the functionality of the transthalamic pathway has been partially corroborated by experiments in the visual system of cats (de Souza et al., 2020). On the other hand, the differential oscillatory response of the neuronal signals between the cortices in certain phases of pulvinar oscillatory activity (Saalmann and Kastner, 2011; Saalmann et al., 2012; Fiebelkorn et al., 2019), as well as when the pulvinar is inactivated (Zhou et al., 2016; Cortes et al., 2020), suggest it plays a role in the effective cortical connectivity. Our results showing bi-stable pulvinar states, suggest that cortical feedback transmission is associated with pulvinar oscillatory activity and the feedforward pathway, with the asynchronous irregular spike response of the pulvinar. Thus, this parallel feedback pathway through the pulvinar would reinforce the top-down attentional activity associated with low-frequency oscillations (*i.e.*, areas 17 and 18). This prediction of feedforward oscillatory activation should be verified in future works, since pulvinar could be indirectly activated by cortico-cortical feedback. That is, from layers 2/3 of a higher cortical area to layer 5 of a lower cortical area, and from here to the pulvinar (e.g., from area 21a to area 17). Such a feedback pathway would induce the type of antagonistic dynamics predicted here (Figure 13).

## Data Availability Statement

The original contributions presented in the study are included in the article/Supplementary Material, further inquiries can be directed to the corresponding author/s.

## Author Contributions

NC, RA, and HL: conceptualization. NC: methodology, software, formal analysis, investigation, data curation, and writing—original draft preparation. NC and CC: validation and visualization. CC: resources, supervision, project administration, and funding acquisition. NC, RA, HL, and CC: writing—review and editing. All authors have read and agreed to the published version of the manuscript.

## Conflict of Interest

The authors declare that the research was conducted in the absence of any commercial or financial relationships that could be construed as a potential conflict of interest.

## Publisher’s Note

All claims expressed in this article are solely those of the authors and do not necessarily represent those of their affiliated organizations, or those of the publisher, the editors and the reviewers. Any product that may be evaluated in this article, or claim that may be made by its manufacturer, is not guaranteed or endorsed by the publisher.
