# Supplementary material for: Corticothalamic Projections Gate Alpha Rhythms in the Pulvinar
Source: Front Cell Neurosci. 2021 Dec 6;15:787170. doi: 10.3389/fncel.2021.787170 (PMC8685293; doi:10.3389/fncel.2021.787170)
Supplement: Supplementary file 1 [file Table_1.DOCX]

**Supplementary Material**

**Supplementary Table 1:** Percentage of low-frequency oscillations for pulvinar and area 21a, during the 3 recording periods of Figure 11.

|  | **Alpha (%)** | ***>*Alpha (%)** | ***<*Alpha (%)** | **Period** |
| --- | --- | --- | --- | --- |
| **Pulvinar** | 99.03 | 0.78 | 0.14 | 1 |
|  | 35.97 | 10.95 | 52.87 | 2 |
|  | 0.00 | 0.00 | 100.00 | 3 |
| **Area 21a** | 64.44 | 0.73 | 34.54 | 1 |
|  | 26.06 | 1.89 | 71.70 | 2 |
|  | 1.69 | 0.03 | 98.29 | 3 |

**Supplementary Table 2:** Values of *η^a17^_1_ and η^a17^_2_ for cases described in Figure 9.*

|  | **Case A** | **Case B** | **Case C** | **Case D** | **Case E** | **Case F** |
| --- | --- | --- | --- | --- | --- | --- |
| ***η^a17^_1_*** | 1.0 | 1.0 | 7.14 | 5.0 | 5.0 | 2.0 |
| ***η^a17^_2_*** | 2.5 | 4.0 | 0.71 | 1.5 | 1.5 | 3.0 |
